# Genetic Variability and Phylogenetic Relationships within *Trypanosoma cruzi *I Isolated in Colombia Based on Miniexon Gene Sequences

**DOI:** 10.1155/2009/897364

**Published:** 2010-02-01

**Authors:** Claudia Herrera, Felipe Guhl, Alejandra Falla, Anabella Fajardo, Marleny Montilla, Gustavo Adolfo Vallejo, M. Dolores Bargues

**Affiliations:** ^1^Centro de Investigaciones en Microbiología y Parasitología Tropical (CIMPAT), Universidad de los Andes, A.A. 4976. Carrera 1a. No. 18-A-10, Bogotá, Colombia; ^2^Grupo de Parasitología, Instituto Nacional de Salud, Avenida calle 26 No. 51-20 - Zona 6 CAN., Bogotá, Colombia; ^3^Laboratorio de Investigaciones en Parasitología Tropical, Facultad de Ciencias, Universidad del Tolima, Ibagué, Colombia. B. Santa Helena A.A. 546, Ibagué, Colombia; ^4^Departamento de Biología Celular y Parasitología, Facultad de Farmacia, Universidad de Valencia, 46100 Burjasot, Valencia, Spain

## Abstract

Phylogenetic studies of *Trypanosoma cruzi* have identified the existence of two groups: *T. cruzi* I and *T. cruzi* II. There are aspects that still remain unknown about the genetic variability within the *T. cruzi* I group. Given its epidemiological importance, it is necessary to have a better understanding of *T. cruzi* transmission cycles. Our purpose was to corroborate the existence of haplotypes within the *T. cruzi* I group and to describe the genetic variability and phylogenetic relationships, based on single nucleotide polymorphisms (SNPs) found in the miniexon gene intergenic region, for the isolates from different hosts and epidemiological transmission cycles in Colombian regions. 31 *T. cruzi* isolates were molecularly characterized. Phylogenetic relationships within *T. cruzi* I isolates showed four haplotype groups (Ia–Id), associated with their transmission cycle. In previous studies, we reported that haplotype Ia is mainly associated with the domestic cycle and domiciliated *Rhodnius prolixus*. Haplotype Ib is associated with the domestic cycle and peridomestic cycle, haplotype Ic is closely related with the peridomestic cycle, and haplotype Id is strongly associated with the sylvatic cycle. The phylogenetic methodologies applied in this study are tools that bolster the associations among isolates and thus shed light on Chagas disease epidemiology.

## 1. Introduction

Chagas disease is a very complex zoonosis and represents a major public health problem in Latin America, affecting about 15 million people, with 28 million people being at risk of acquiring the infection. It has been estimated that nearly 5% of the Colombian population is infected and that 20% are at risk of acquiring the disease [1]. The infection, caused by the protozoan *Trypanosoma cruzi*, affects people during acute and chronic phases with different degrees of severity. The acute stage appears shortly after the infection and the chronic stage may last several years. After a silent asymptomatic period lasting several years, about 25% of the patients develop cardiac symptoms that may lead to chronic heart failure and sudden death, 6% develop digestive lesions, mainly megacolon and megaesophagus, and 3% suffer peripheral nerve damage [2]. These manifestations are not equally distributed within countries [3]. 

As a result of their particular method of clonal propagation, *T. cruzi* isolates have been described as showing great phenotypical and genotypical heterogeneity [4, 5]. Despite their high genetic variability, *T. cruzi* isolates can be classified into two groups, *T. cruzi* I and *T. cruzi* II [6], the latter being divided into five subgroups IIa–e [7], whereas *T cruzi* I consists of a single, relatively homogeneous clade [8]. 

*T. cruzi *I, which has a very large geographical distribution from North to South America, predominates from the Amazonian basin northwards, where domestic and sylvatic triatomine species ensure the transmission of Chagas disease in various countries, including Venezuela, Colombia, Central America, and Mexico [9–13]. In the southern cone, countries such as Bolivia, Argentina, and Chile have reported the presence of *T. cruzi* I circulating in domestic and sylvatic transmission cycles [3, 14, 15]. 

Intraspecific variation within *T. cruzi* I has been extensively documented by molecular inferences and biological characterization. In recent years, O'Connor et al. have published studies about genetic variability in *T.cruzi* I [9] and our group proposed the existence of four haplotypes within *T. cruzi* I [16]. The inferences described in our study were based on polymorphisms found in the miniexon gene's intergenic partial region. This gene is involved in posttranscriptional events, such as mRNA processing, and it has been proposed as an important molecular marker due to its essential role as a control mechanism of differential protein expression and its high variability within *T. cruzi *populations [17–19].

 The identification and characterization of *T. cruzi* I haplotypes would be a good epidemiological tool for the understanding of transmission dynamics in endemic areas of Latin America. With this study, we show how the previously proposed haplotypes, based on specific polymorphisms in the partial intergenic region of the miniexon gene that are related to the parasite's transmission cycles, are found in other Colombian isolates and can be usefull to identified other isolates from Latin America.

## 2. Materials and Methods

### 2.1. *T. cruzi* Isolates

A total of 31 *T. cruzi* isolates were evaluated from different hosts and Colombian regions. Characterized *T. cruzi* I and *T. cruzi* II isolates were used as controls (Table 1).

### 2.2. Sequencing of the Miniexon Gene Intergenic Region

DNA was obtained from parasites maintained in culture using the QIAmp DNA extraction kit (Qiagen) and subsequently stored at −4°C. Characterization of the miniexon was performed using previously described primers [22]. PCR was performed on a 20 *μ*L reaction mixture containing 30 ng/*μ*L DNA, 2.5 mM MgCl_2_, 0.2 *μ*M of each primer, and 0.5 U/*μ*L of BIOTOOLS DNA Polymerase. Amplification products were generated in a PT-150 Minicycler (MJ Research, Watertown, MA, USA) by 27 cycles of 30 seconds at 94°C, 30 seconds at 55°C, and 30 seconds at 72°C, followed by a final elongation of 5 minutes at 72°C. Primers and nucleotides were removed from PCR products by purification using the Ultra Clean PCR Clean-up DNA Purification System (MoBio, Solana Beach, CA, USA) according to the manufacturer's protocol and resuspended in 50 mL of 10 mM TE buffer (10 mM Tris-HCl, 1 mM EDTA, pH 7.6). DNA purity and concentration were determined using an Eppendorf Biophotometer 6131 at 260/280 nm wavelengths. 

Sequencing was performed on both strands by the dideoxy chain-termination method and the Taq dye-terminator chemistry kit for the ABI 3730 and ABI 3700 capillary system (Perkin Elmer, Foster City, CA, USA). Multiple alignments were performed using the ClustalW application v.1.8 [23] with default parameters. The alignment was edited in MEGA Version 3.0 [24] and the Staden Package Version 1.5 [25].

#### 2.2.1. Phylogenetic Inference

Phylogenies were inferred using Maximum Parsimony (MP) and Maximum-Likelihood (ML) estimated with PAUP v.4.0b10 [26]. MP analysis was performed using a heuristic algorithm. To assess the relative support of internal nodes, a bootstrap-resampling approach (with 1000 replicates) was performed. 

The alignment of the 32 sequences corresponding to *T. cruzi* I (including the* T. cruzi *I sequence used as a control) was evaluated to obtain the best-fit model in MrModel Test 2.2 [27], a modified version of model test 3.6 [28] and by the Akaike Information Criterion (AIC) [29, 30]. An analysis of MP was performed using a heuristic search, adding 10 sequences randomly per cycle.

The ML method was performed based on the maximum parsimony results, and according to these data, maximum-likelihood trees were reconstructed by TreeView (Win32) v1.6.6. The robustness of the nodes was evaluated by bootstrap on 1000 replications with heuristic research [31]. Bayesian inference of phylogeny was made using MrBayes v 3.1 [32] (settings according to MrModel test 2.2), 10 million Monte Carlo Markov chain generations (Bayesian-Monte Carlo simulation by MrBayes sampling every 100 simulations, burn-in 10000, number of chains 4).

## 3. Results

### 3.1. Miniexon Gene Intergenic Region Sequences

A length of 319 bp was obtained in all isolates for the miniexon SL region gene (spliced leader region). In the alignment of sequences, there were 263 constant sites and 56 parsimony informative sites. A total of 19.43% polymorphic sites were observed, which corresponded to 23 transitions, 14 transversions, and 19 insertion-deletions. The average nucleotide composition (58.4%) was based on GC content (Table 2). The sequences obtained were deposited in GenBank-EMBL-DDJB databases (accession numbers AM259467-AM259480, EU127299-EU127315, EU344771, and EU344772). 

**Figure 1:**
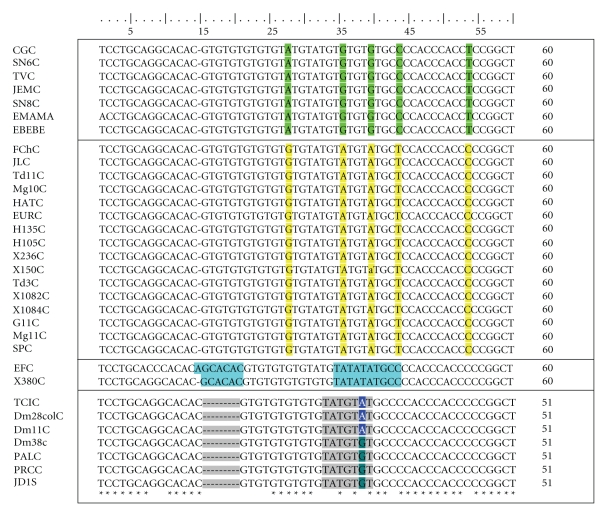
A microsatellite region with an average of 56 nt in which the variability between the 4 proposed haplotypes can be clearly observed. Alignment of the miniexon gene from *T. cruzi *strains. *Differences are not observed in the alignment. The boxed area corresponds to the nucleotide polymorphisms (SNPs) found between the four proposed haplotypes.

The isolates were clustered into four haplotype groups based on the polymorphic sites in the initial region composed of 56 nucleotides (nt) of the no transcriptional miniexon gene, which showed a continuous microsatellite region (TG)n (TATG)m (TG)x. The number of different repetitions varied between 4 and 7 (n), 2 and 4 (m) and 0 and 3 (x) (positions 15–40) (Figure 1).

### 3.2. Phylogenetic Analyses Inferred from Partial Miniexon Gene Intergenic Region Sequences

**Figure 2:**
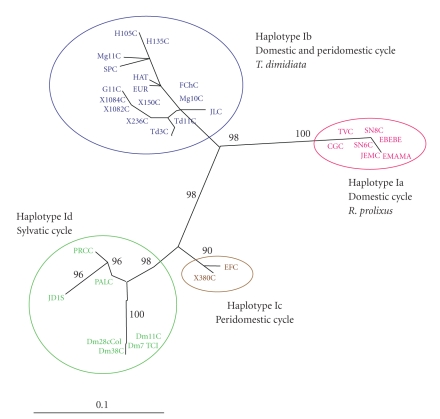
Unrooted phylogenetic tree depicting the evolutionary relationships among *T. cruzi *I isolates. The tree was constructed by the maximum likelihood method. Numbers in larger fonts represent the bootstrap values for the main cluster of the haplotypes. The topology obtained for this method represents the consensus from 236 trees (100 replicates each producing, on average, 4 most parsimonious trees), bootstrap 50% majority rule consensus.

The best ML model fitting the intergenic region data was GTR+G+I. The ML tree obtained (-lnL = 900.8816; proportion of invariable sites = 0.560; gamma distribution shape parameter = 0.893) clearly corroborated the existence of four well-supported haplotypes within the *T. cruzi *I isolates analyzed and their relation with the transmission cycle (Figure 2). 

**Figure 3:**
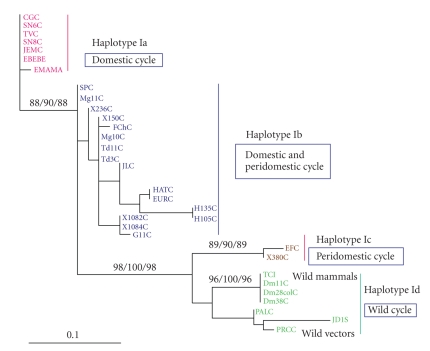
Unrooted phylogenetic tree depicting the evolutionary relationships between *T. cruzi *I isolates. Phylogram showing support nodes from Bayesian-estimated likelihood with 10 million Monte-Carlo Markovian chain generation node support from the >50%-majority rule consensus GTR+I+G model. Invariant sites and gamma distribution values are at the right side of the slash (MP bootstrap values/Bayesian probabilities × 100/ML bootstrap values).

The ML analyses showed four clusters supported by significant bootstrap values (>95%). In Figures 2 and 3, the phylogenetic analyses showed haplotype Id, corresponding to isolates from* Didelphis marsupialis* and wild triatomines, with 96% bootstrap support and 100% Bayesian probabilities. The haplotypes Ia, Ib, and Ic were supported with 98% bootstrap. Haplotypes Ia and Ib corresponded to the domestic and peridomestic cycles, respectively, showing a bootstrap value of 98%, and they were more related in comparison with haplotype Ic. Haplotype Ic was formed by isolates from the peridomestic cycle; however, although they belonged to this cycle, they were closely related to haplotype Id (sylvatic cycle), with a bootstrap value of 98%. 

Haplotype Id was mainly related with the sylvatic environment (isolates obtained from *D. marsupialis* and *Rhodnius prolixus *sylvatic population) supported by a significant bootstrap value of 96% (Figure 3).

A MrBayes credibility method was used to give statistical validity. The Bayesian probabilities (>95%) showed three significant clades: one formed by the domestic isolates, haplotype Ia, which showed a strong polytomy, a second clade (Ib) with a Bayesian probability of 100% formed by isolates from domestic and peridomestic cycles, and a third main clade formed in which haplotypes Ic and Id were closely related. However, these isolates presented punctual mutations that allowed us to group them in different haplotype groups.

## 4. Discussion

*T. cruzi *I isolates have been found mainly associated with the sylvatic transmission cycle [33, 34] and parasites isolated from marsupials [35, 36]. *T. cruzi *II has been mainly associated with the domestic transmission cycle and infection in primates [33]. This has led to the misconception that *T. cuzi *I is always associated with the sylvatic cycle of transmission [8]. Recent epidemiological studies performed in the northern regions of South America, in Central America, and Mexico have demonstrated a predominance of *T. cruzi* I in the sylvatic cycle of transmission, as well as in the domestic and peridomestic cycles [11, 12, 37]. 

The miniexon gene has been widely used as a taxonomic marker due to its high heterogeneity and the presence of large multicopy genomic arrays in the parasite genome [16, 17]. The haplotypes within the *T. cruzi* I group of Colombia have been identified from phylogenetic analyses such as MP, ML, and BI as follows: haplotype Ia, associated with the domestic cycle and the vector *R. prolixus*; haplotype Ib, associated with the domestic and peridomestic cycles and the vector *Triatoma dimidiata*; haplotype Ic, associated with the peridomestic cycle; and haplotype Id, associated with the sylvatic cycle of transmission. 

Haplotype Ib is associated with humans, peridomestic vectors (*T. dimidiata*), and isolates from reservoirs associated with the peridomicile (*Canis familiaris*). Haplotype Ic is not clearly defined within a specific transmission cycle and seems to play an intermediary role between the domestic and sylvatic cycles. Furthermore, there is a phylogenetic association with haplotype Id that is supported by a bootstrap of 98%, grouping the isolates in a monophyletic clade (Figures 2 and 3). To confirm this observation requires a larger sample of isolates for either defining haplotype Ic as an independent group or classifying it into sylvatic haplotype Id [16, 18].

The isolates from wild *R. prolixus* captured in palms (*Attalea butyracea*) and the wild reservoir *D. marsupialis* have been grouped in haplotype Id. Phylogenetic analysis shows a strong group (haplotype Id), which includes the isolates with sylvatic origin, and the bootstrap value (96%–100%) statistically supports this group. This result also corroborates the observation by O'Connor et al. [9] in which the “*Didelphis-group*” was proposed, adding in from the present study the isolates from sylvatic vectors. We found a subdivision, based on nucleotide polymorphisms at position 39, in haplotype Id. This small difference linked to the wild origin of the vectors found in this group and was not considered as a fifth haplotype (Figure 1). 

The proposed haplotypes have a direct association with the epidemiologic cycle from which they come, but there was not a direct relationship with the geographical isolation. The variability found between the haplotypes can be explained by the diverse fauna in different regions and by clearance processes that are occurring as environmental changes are occurring. Unfavorable changes and the subsequent rarefaction that occurs in wild fauna favor the overlapping of transmission cycles and the consequent molecular diversification of parasite strains [38].

This phenomenon is influenced by environmental and immunological factors, including virulence, pathogenicity, and possible selection of strains and clones after interacting with vectors and vertebrate hosts. The combination of several of these factors might explain the variability in the biological behavior of the parasite [39].

The primitive transmission was restricted to tropical forest environments, where the parasite spread initially by anal gland secretions and urine from the didelphides [40, 41]. Different genetic characterizations performed on *T. cruzi* I isolates have suggested a strong association with *Didelphis *spp., while at the same time, there is an association of* T. cruzi *II with armadillos [8]. Interestingly, *R. prolixus* has been related to mammals in palms within wild ecotopes. In addition, these relationships were observed throughout North and South America [42].

The opossum genus presents the highest distribution in the world and ranges found from southern Canada to southern Argentina. *D. albiventris* lives preferentially in the cold climates of South America. *D. marsupialis* inhabits warm forest areas, mainly in the northern part of South America and Central America, where they maintain the same distribution as *T. cruzi *I [9].

The association of *T. cruzi *I with *Didelphis *spp. has also been observed in the United States, where *D. virginiana* migrated to North America during the Pleistocene approximately 40 million years ago [9, 43, 44]. The molecular characterization of parasites isolated from *D. albiventris* has also demonstrated the presence of *T. cruzi *I. It has been demonstrated that phylogenetic groups of *T. cruzi* infect certain hosts in a preferential manner. Furthermore, there is evidence that *T. cruzi* II produces high parasitemia in rodents, while in *D. marsupialis*, *T. cruzi* II is eliminated and *T. cruzi* I is withheld [45]. 

The relationship between the sylvatic and domestic cycle is extremely complex; however, a common feature in the two cycles is the presence of didelphydes, which allegedly play an important role in the dynamics of transmission [46]. The coevolution of *T. cruzi *I and *D. marsupialis *suggests an ancestral and parental relation that has produced all genetic subdivisions of the parasite identified in this study. This reported variability has favored the parasite's dispersion from the insect to wild mammals to domestic mammals [8, 16, 33, 34, 47].

Based on these observations, we suggest that *T. cruzi *I isolates are part of an ancestral group in America beginning with the sylvatic cycle of transmission between vectors and wild reservoirs, in which *D. marsupialis* acted as a transmission bridge between the domiciliary and peridomiciliary cycles, given its ability to shift from domestic to wild environments where this marsupial sometimes goes in search of food [33, 48].

Different studies have reported the vector *R. prolixus* as a factor for the final selective transmission of *T. cruzi* I for humans and reservoirs. This suggests that these sylvatic *R. prolixus*, widely distributed in palm trees [49, 50], may have begun the process of the passage of *T. cruzi* I from the wild cycle to a domestic cycle at the time they began their domiciliation process [49, 51].

These results support, for the first time, a very close relationship between *T. cruzi* I haplotypes Ib and Id and vectors *R. prolixus* and *T. dimidiata*, respectively. Previous studies, with an emphasis on the role of vectors in the transmission of trypanosome subpopulation, have shown a close correlation between genotypes of *T. rangeli* and *Rhodnius *species [51–54].

The trypanosome interaction with different triatomine species and populations is apparently an important factor determining the trypanosome epidemiology that infects humans in Latin America. Frequently, specific subpopulations of trypanosomes are transmitted by specific vectors in a particular geographic area.

Future studies focused on the trypanosome-vector interaction will allow the identification of coevolutionary processes, which in turn could support the evolutionary hypothesis and the distribution of *T. cruzi* and *T. rangeli* vectors in America. Additionally, these studies will help to identify the mechanisms that facilitate or impede the transmission of parasites by different vector species [43]. However, to establish a relationship between the different *T. cruzi* I haplotypes, further analyses using additional suitable molecular markers, such as mitochondrial and flagellar genes, are required.

## Figures and Tables

**Table 1:** Geographical origin, host, reservoir, and cycle of the *T. cruzi* isolates analyzed from different parts of Colombia with *T. cruzi* II used as a control.

Code Isolates	Abbreviated code	Host/Vector	Geographical origin	Cycle
MHOM/CO/03/CG	CGC	Human (acute phase,VIH)	Caqueta	Domestic
MHOM/CO/92/FCH	FChC	Human (acute phase)	Norte de Santander	Domestic
MHOM/CO/JEM	JEMC	Human (acute phase)	Putumayo	Domestic
MHOM/CO/92/JL	JLC	Human (acute phase)	Arauca	Domestic
MHOM/CO/SP	SPC	Human (acute phase)	Casanare	Domestic
MHOM/CO/07/EB [20, 21]	EBEBE	Human (Congenital)	Boyacá	Domestic
MHOM/CO/07/EMA [20, 21]	EMAMA	Human (acute phase)	Boyacá	Domestic
MCanis/CO/H135	H135C	*Canis familiaris*	Boyacá	Domestic
MCanis/CO/H105	H105C	*Canis familiaris*	Boyacá	Domestic
MDID/CO/28	Dm28C	*Didelphis marsupialis*	Tolima	Sylvatic
MDID/CO/11	Dm11C	*Didelphis marsupialis*	Tolima	Sylvatic
MDID/CO/38	Dm38C	*Didelphis marsupialis*	Tolima	Sylvatic
IRHO/CO/SN6	SN6C	*Rhodnius prolixus*	Magdalena	Domestic
IRHO/CO/SN8	SN8C	*Rhodnius prolixus*	Magdalena	Domestic
IRHO/X/CO/380	X380C	*Rhodnius prolixus*	Boyacá	Domestic
IRHO/CO/X236	X236C	*Rhodnius prolixus*	Boyacá	Domestic
IRHO/CO/X150	X150C	*Rhodnius prolixus*	Boyacá	Domestic
IRHO/CO/X1082	X1082C	*Rhodnius prolixus*	Boyacá	Domestic
IRHO/CO/X1084	X1084C	*Rhodnius prolixus*	Boyacá	Domestic
IRHO/CO/PAL	PALC	*Rhodnius prolixus *	Casanare	Sylvatic
IRHO/CO/JDI	JD1S	*Rhodnius prolixus *	Vichada	Sylvatic
IRHO/CO/03/PRC	PRC	*Rhodnius prolixus *	Caqueta	Sylvatic
IRPallescens/CO/Mg11	Mg11C	*Rhodnius pallescens *	Magdalena	Peridomestic
ITr dimidiata/CO/Mg10	Mg10C	*Triatoma dimidiata*	Magdalena	Peridomestic
ITr dimidiata/CO/Td11	Td11C	*Triatoma dimidiata*	Boyacá	Peridomestic
ITr dimidiata/CO/Td3	Td3C	*Triatoma dimidiata*	Boyacá	Peridomestic
ITri dimidiata /CO/HAT	HAT	*Triatoma dimidiata*	Boyacá	Peridomestic
ITri dimidiata /CO/EUR	EUR	*Triatoma dimidiata*	Boyacá	Peridomestic
ITri dimidiata /CO/EF	EFC	*Triatoma dimidiata*	Boyacá	Peridomestic
ITri dimidiata /CO/G11	G11C	*Triatoma dimidiata*	Boyacá	Peridomestic
ITr venosa/CO/04/TV	TVC	*Triatoma venosa*	Boyacá	Peridomestic
MDID/CO/7	Dm7^(a)^	*Didelphis marsupialis*	Tolima	Sylvatic
MHOM/Br/167	Tc 167^(b)^	Human	Minas Gerais Brasil	Domestic

^a,b^ DNA obtained from previously characterized strains (as controls).

**Table 2:** Length, GC content (%), and GenBank accession numbers for reported sequences of *T. cruzi *I and *T. cruzi *II isolates.

TCI isolates	% GC	Bp length	GB accession No.
CGC	58.7	313	AM259467
JLC	58.9	314	AM259468
FChC	58.8	314	AM259469
JEMC	58.7	313	EU127299
Dm28colC	57.7	303	AM259470
EBEBE	58.7	314	EU344771
EMAMA	58.7	314	EU344772
SN6C	58.9	312	AM259471
X380C	58.3	310	AM259472
PAL C	59	303	AM259473
EFC	57.1	313	AM259474
Td 11C	58.6	312	AM259475
TVC	58.5	314	AM259476
Mg10C	57.8	311	AM259477
JD1S	58	305	AM259478
SPC	58	314	EU127300
H135C	58.2	316	EU127301
H105C	58.2	316	EU127302
Dm38C	58	305	EU127303
Dm11C	58	305	EU127304
SN8C	58.7	313	EU127305
X236C	58.9	314	EU127306
X150C	58.9	314	EU127307
X1082C	58.2	314	EU127308
X1084C	58.2	314	EU127309
PRCC	58.6	314	EU127310
Mg11C	58.9	314	EU127311
Td3C	58.6	314	EU127312
HATC	58	314	EU127312
EURC	58	322	EU127314
G11C	58.6	314	EU127315
TCIC	58.2	304	AM259479
TCIIC	58	298	AM259480
